# Anatomical and biometric study of the mitral valve apparatus: application in valve repair surgery

**DOI:** 10.1186/s13019-023-02232-2

**Published:** 2023-04-14

**Authors:** Misbaou Barry, Mesut Gun, Yuthiline Hun-Chabry, Majid Harmouche, Johann Peltier, Thierry Caus, Eric Havet

**Affiliations:** 1 Laboratory of Anatomy, Faculty of Medicine, University of Picardie-Jule Vernes, Amiens, France; 2 Department of Cardiac Surgery, Amiens Picardie University Hospital Center, 1 Rue du Professeur Christian CABROL, 80054 Amiens Cedex1, France; 3 Department of Cardiology, Amiens Picardie University Hospital Center, 1 Rue du Professeur Christian CABROL, 80054 Amiens Cedex 1, France; 4 Department of Radiology, Amiens Picardie University Hospital Center, 1 Rue du Professeur Christian CABROL, 80054 Amiens Cedex 1, France

**Keywords:** Mitral valve repair, ePTFE neocording, Biometry, Main mitral valve cordaes, Papillary muscles

## Abstract

**Objective:**

Most mitral valve repair techniques provide excellent surgical results by removing regurgitation, but all of these techniques simultaneously reduce posterior valve mobility. A comprehensive biometric study of the mitral valve apparatus will provide landmarks that would help improve this posterior valve mobility.

**Materials and methods:**

Thirty one (31) human hearts have been studied, from 14 women and 17 men. The characteristics of the studied sample were analyzed descriptively. The difference in means of the variables between women and men were tested using a Student t test. Correlations between the different measures were determined by simple regression analysis. Mean values are shown with ± 1 standard deviation and the limit of significance was set at 0.05.

**Results:**

The mean weight of the hearts was 275.3 ± 2.4 g. The anteroposterior diameter of the mitral annulus was 29.3 ± 1.22 mm, the intertrigonal distance was 25.2 ± 3.50 mm and the anterior leaflet to posterior leaflet ratio was 1.9 ± 0.10, the length of the chordae A2 = 19.4 ± 1.15 mm and P2 = 14.5 ± 0.85 mm. The length of the anterior papillary muscle averaged 30.9 ± 7.20 mm and that of the posterior one 30.0 ± 8.75 mm. The comparison of the different values measured between women and men showed no statistically significant difference (*p* > 0.05). There was no correlation between these different measured values (*p* > 0.05).

**Conclusion:**

A perfect knowledge of anatomy and biometry is therefore essential to offer alternative techniques that reproduce the real anatomy and physiology with a complete reconstruction of the mitral valve.

## Introduction

The first person to describe the mitral valve in detail was Leonardo da Vinci in the late 1400's. Subsequently it was Andreas Vesalius (1514–1564), reported by [1], who gave it its name. He compared the shape of the two valvular leaflets to a bishop's headdress "the mitre". Hence the adjective mitral which characterises the valve. It was W. Harvey (1578–1657), according to [2], who clarified his physiology by describing the blood circulation in 1628. It was in 1957 that CW LILLEHEI published his results [3], on the initiation of the mitral valve repair surgery. Since the work of Alain CARPENTIER, in 1969 which were published by his team [4], it has benefited from considerable progress. Multiple techniques reported by several authors [5–11], have been used (quadrangular resection followed by plication of the annulus or triangular resection of the proclaimed middle part of the small valve, transposition of cordaes, neo-cording, enlargement patches, flexible rings, partial homogenization, etc.). All of these techniques offer excellent surgical results by eliminating regurgitation, but all of these techniques reduce posterior valve mobility at the same time.

We wanted to understand if it is possible, based on a complete study of anatomy and biometry, to have a procedure that would allow to preserve the movement of the valve leaflets after a repair plasty.

The objective of this article is to undertake a comprehensive biometric study of the main chordaes (which are ruptured or elongated in mitral insufficiency), the mitral annulus and the papillary muscles in the human heart, to look for benchmarks that would help surgeons to improve their techniques, to determine if the average of the measurements in women is different from that of men, and to look for a correlation between the different values measured as well as for a correlation between age and each of these values.

## Materials and methods

Thirty one (31) human hearts have been studied, from 14 women and 17 men, with a mean age of 79 ± 10 and extremes of 50 and 97 years old. Each of these hearts was fixed with 10% formalin. Prior to examination of each heart, the aorta and pulmonary artery were resected 1 cm above the valve commissure. The resection was also performed at the foot of the superior and inferior vena cava and each heart was weighed. To verify the absence of mitral regurgitation, each valve was inspected through the left atrium opened at its left edge, while saline solution was injected under pressure through the aorta. Then the left border of the left ventricle was also opened to reveal the mitral annulus, anterior valve, posterior valve, chordaes and papillary muscles. These different elements of the mitral valve were examined and the main chordaes were counted per valve. The following measurements were carried out:

1. the antero-posterior diameter which goes from the middle of the anterior mitral annulus (base of implantation of the anterior leaflet) thus passing through the center of A2 and P2 to that of the posterior ring (base of implantation of the posterior leaflet).
2. The inter-commissural diameter of the mitral ring which goes from the anterior commissure (limit between A1 and P1) to the posterior commissure (limit between A3 and P3).
3. The intertrigonal distance which goes from the posterior or right trigone (dense junction zone located between the mitral valve, the aortic valve and the tricuspid valve overhanging the fibrous septum trigone) to the anterior or left trigone (located at the junction of the two left fibrous edges aorta and mitral valve). A fibrous part connects the two trigones on which is inserted the mitral ring (base of the anterior leaflet).
4. The length of the anterior leaflet which goes from the middle of the anterior mitral annulus (base of implantation of the anterior leaflet) to the free edge of the anterior leaflet, thus passing through the center of A2.
5. The length of the posterior leaflet which goes from the middle of the posterior mitral annulus (base of implantation of the posterior leaflet) to the free edge of the posterior leaflet, passing through the center of P2.
6. The length of the marginal cordaes which goes from the point of the head of the papillary muscle concerned to the free edge of the valve.
7. The length of the papillary muscles which goes from the base of implantation of the papillary muscle on the ventricle to the top of the head of the papillary muscle concerned.
8. 8-The distance between the papillary muscles and the annulus which goes from the top of the head of the papillary muscle concerned to the mitral annulus.
9. The distance between the papillary muscles and the apex which goes from the base of implantation of the papillary muscle concerned on the ventricle to the apex.

**Fig. 1:**
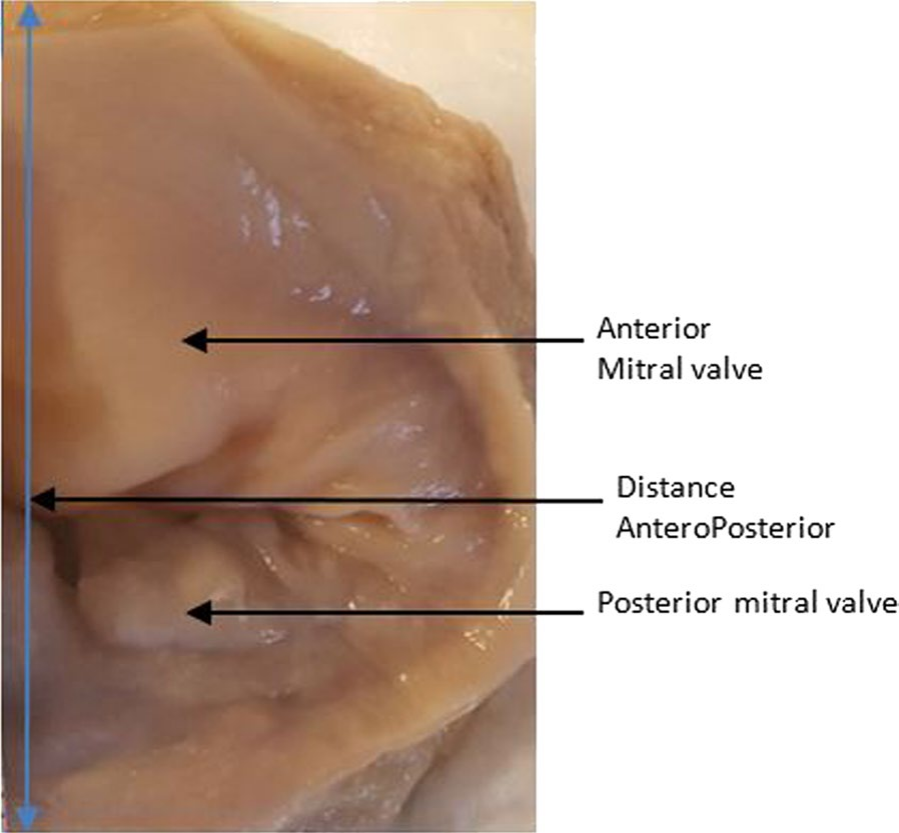
Anterior valve, posterior valve and anteroposterior distance of mitral valve

**Fig. 2:**
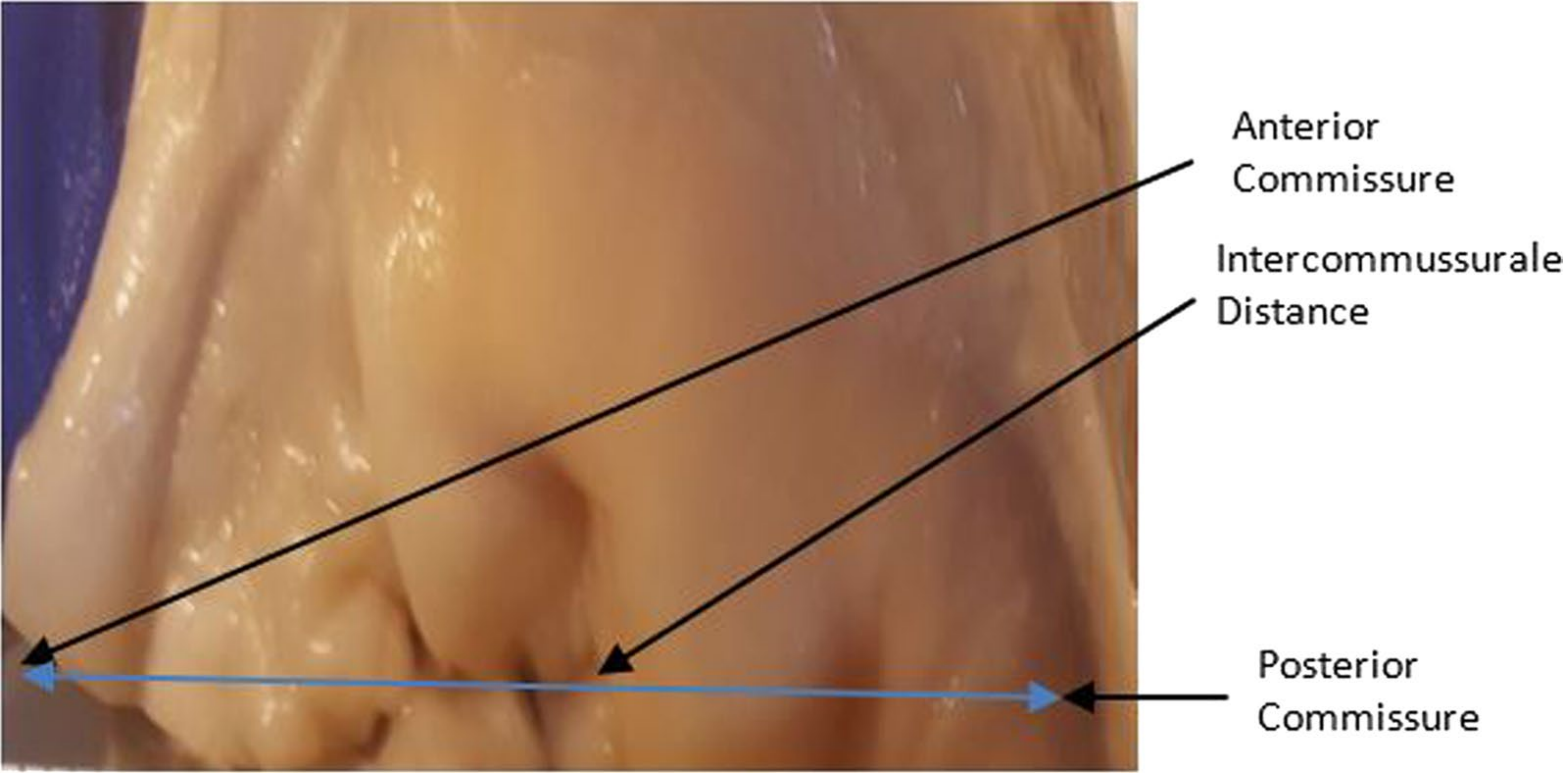
Anterior commissure, posterior commissure and intercommussural distance

**Fig. 3:**
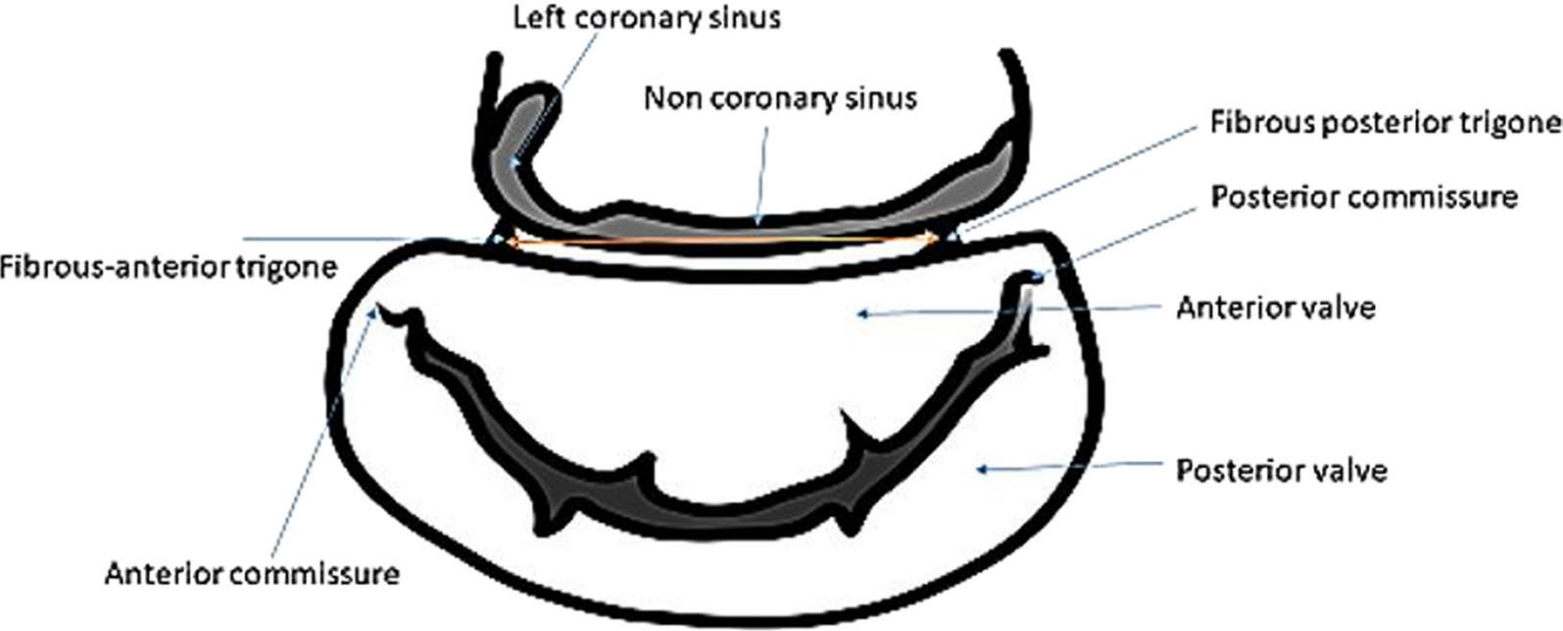
Representation of the distance between two trigons

**Fig. 4:**
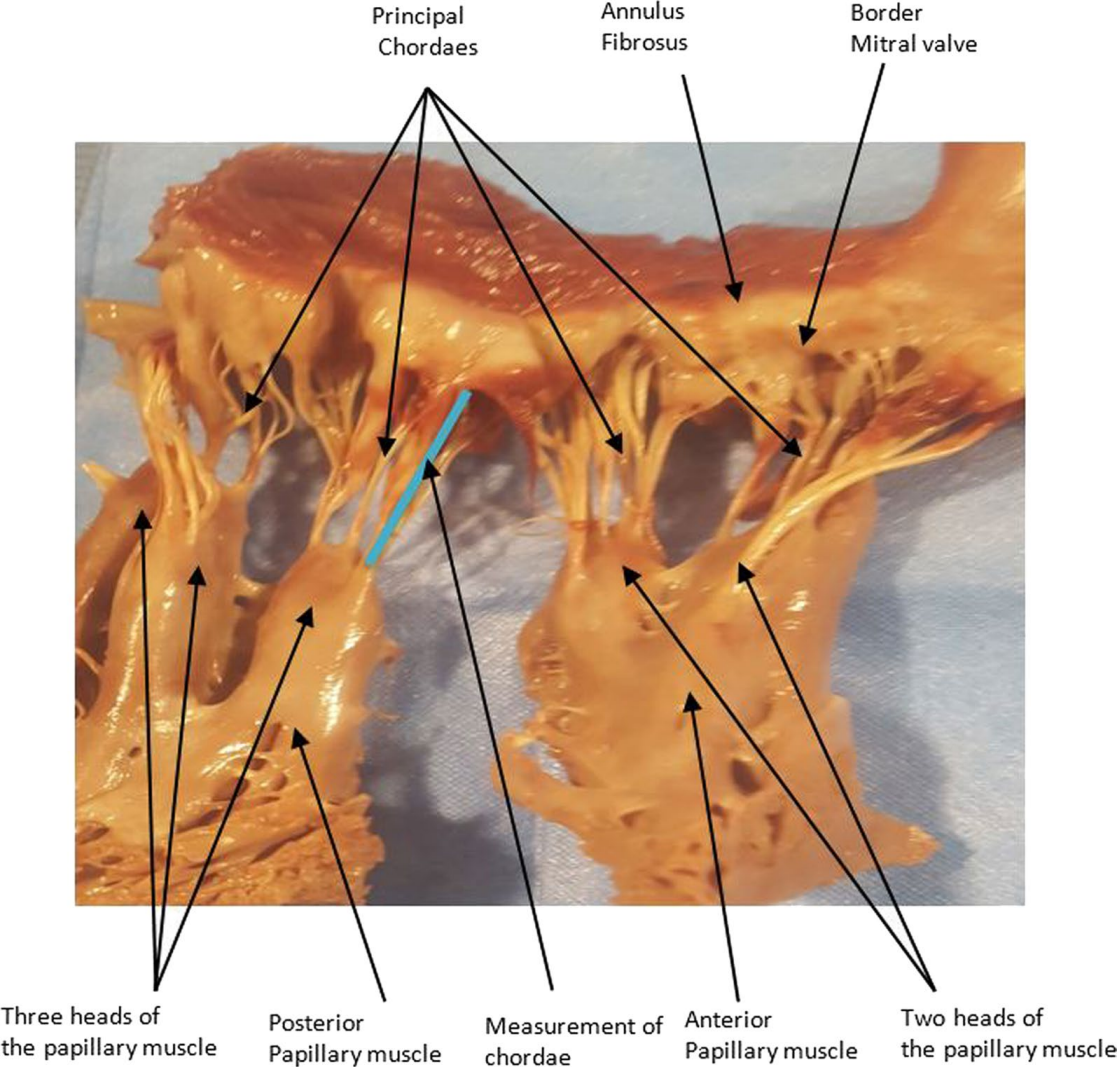
The different elements of the valve (principal chordae, anterior and posterior papillary muscles, papillary muscle heads, annulus and edges of mitral valves)

**Fig. 5:**
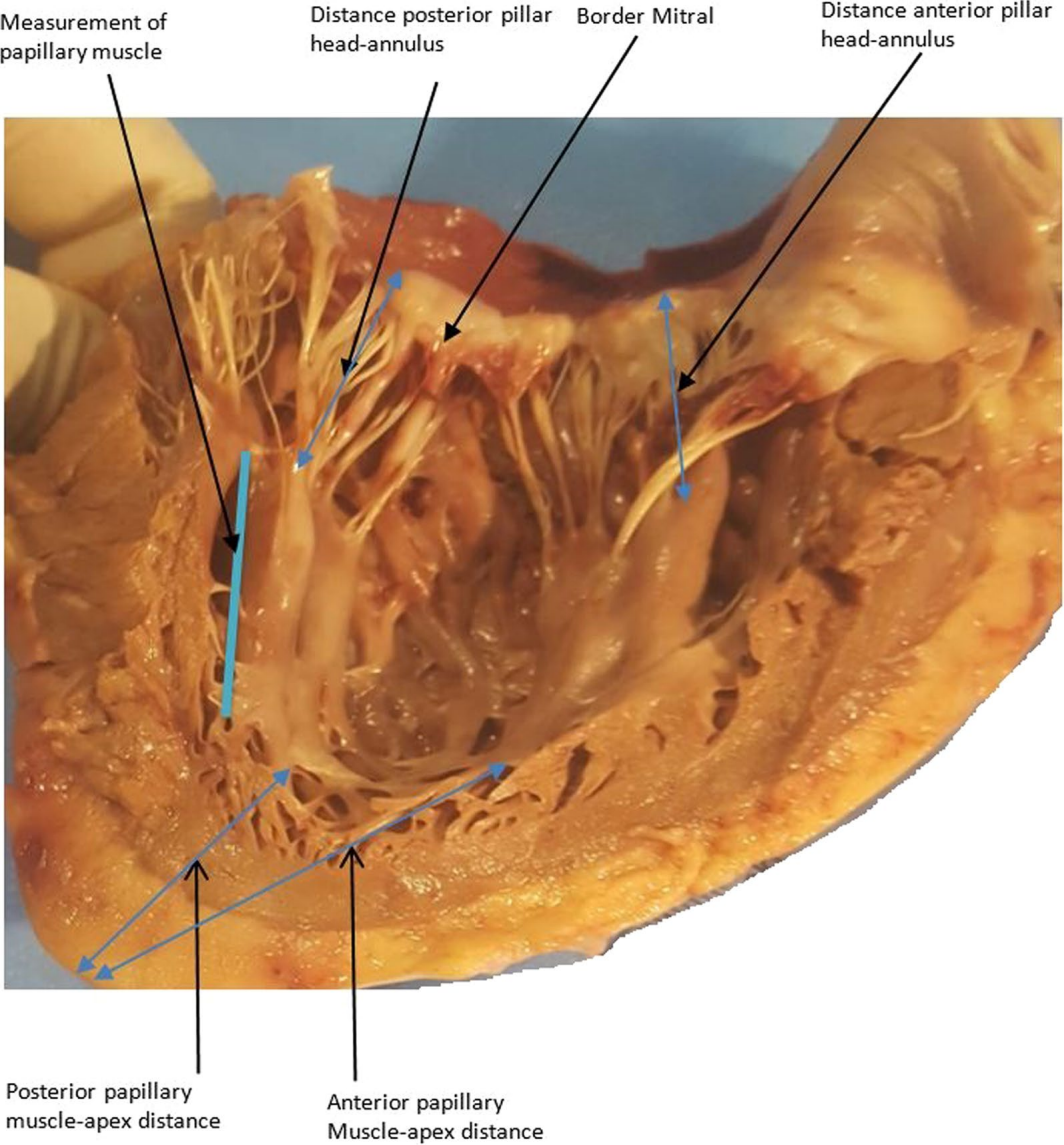
The distance between the papillary muscles and the annulus as well as the distance between the papillary muscles and the apex

The number of heads per papillary muscle and the number of chordae per papillary muscle were also counted. See Figs. 1, 2, 3, 4 and 5. All measurements were made with a RISEMART digital caliper with a precision of 0.2 mm. Three successive measurements allowed to have an average of measurement. The measurements were made by a single operator accompanied by an assistant.

## Statistical method

Sample characteristics were analyzed in a descriptive way, which allowed us to collect means and standard deviations for the quantitative variables. The normality of the chord lengths was verified by the Shapiro–Wilk test, Concerning gender, the difference in the means of the variables between women and men were tested using a Student t-test. Correlations between the different measures were determined by simple regression analysis. Mean values are shown with ± 1 standard deviation, and the limit of significance was set at 0.05.

Statistical analysis was performed using SAS software version 7.1.

*Terminology* The three segments of the anterior leaflet are named: A1 (anterior), A2 (median) and A3 (posterior). The three segments of the posterior leaflet has the opposite segments P1 (anterior), P2 (median) and P3 (posterior), A (anterior valve), P (posterior valve).

## Results

The aortic annulus was oval in shape, flattened in its portion adjacent to the aortic annulus between the two fibrous trigons in all our patients. Its anteroposterior diameter averaged 29.3 ± 1.22 mm and its intercommensural diameter averaged 33.8 ± 1.37 mm. The intertrigonal distance measures 25.2 ± 3.50 mm and the anterior to posterior leaflet ratio was 1.9 ± 0.10 (Table 1).

**Table 1:** Measurements of the mitral ring, intertrigonal distance, and anterior to posterior leaf ratio (n = 31)

Variable	Mean ± SD
Anteroposterior diameter (mm)	29.3 ± 1.22
InterCommensural diameter (mm)	33.8 ± 1.37
InterTrigonal diameter (mm)	25.2 ± 3.50
A2 (mm)	17.0 ± 0.63
P2 (mm)	9.0 ± 0.63
P2/A2	0.5 ± 0.10

The mean weight of the hearts was 275.3 ± 2.41 g. Tendon chordaes connect the mitral leaflets to both papillary muscles. The length of the chordaes differed according to their position. The average length of the chordaes at the anterior valve was A1 = 17.5 ± 1.12 mm, A2 = 19.4 ± 1.15 mm, A3 = 17.6 ± 1.09 mm. At the level of the posterior valve the chordae measures on average: P1 = 13.3 ± 0.83 mm, P2 = 14.5 ± 0.85 mm, and P3 = 13.3 ± 0.89 mm. Anterior commissure chordaes measured an average of 13.2 ± 0.82 mm and posterior commissure chordaes measured 15 ± 1.17 mm. For the anterior valve, the number of chordaes is 3.0 ± 0.68 at A1, at A2 it is 3.4 ± 0.50, and at A3 it is 3.0 ± 0.00. For the posterior valve, the number of chordaes is 2.8 ± 0.43 at P1, 3.6 ± 0.50 at P2, and 3.2 ± 0.40 at P3 (Table 2).

**Table 2:** The number and length of principal chordae per valve (n = 31)

Variable	Mean (SD)
Heart weight (g)	277.74 (25.65)
Chordae length P2 (mm)	14.52 (0.85)
Chordae length P3 (mm)	13.26 (0.89)
Chordae length A1 (mm)	17.45 (1.12)
Chordae length A2 (mm)	19.42 (1.15)
Chordae length A3 (mm)	17.58 (1.09)
Anterior commissure chordae length (mm)	13.16 (0.82)
Posterior commissure chordae length (mm)	14.97 (1.17)
Number chordae A1	3.00 (0.68)
Number chordae A2	3.39 (0.50)
Number chordae A3	3.00 (0.00)
Number chordae P1	2.77 (0.43)
Number chordae P2	3.61 (0.50)
Number chordae P3	3.19 (0.40)
Total number chordaes	18.97 (0.98)

The length of the anterior papillary muscle is 30.9 ± 7.20 mm and that of the posterior papillary muscle is 30.0 ± 8.75 mm. The number of heads for the anterior papillary muscle is 1.7 ± 0.65 and for the posterior papillary muscle it is 1.5 ± 0.68.

The number of chordaes that insert at the anterior papillary muscle is 11.7 ± 2.18 and it is 9.6 ± 1.99 at the posterior papillary muscle. The distance between the head of the anterior papillary muscle and the mitral annulus is 21.9 ± 7.25 mm, and that between the head of the posterior papillary muscle and the mitral annulus is 21.0 ± 5.40 mm. The distance from the head of the anterior papillary muscle to the apex is 18.7 ± 5.29 mm and that from the head of the posterior papillary muscle to the apex is 18.3 ± 4.58 mm (Table 3).

**Table 3:** The morphological characteristics of the papillary muscles (n = 31)

Variable	Mean ± SD
Anterior papillary muscle length (mm)	30.9 ± 7.20
Number of heads of the anterior papillary muscle (mm)	1.7 ± 0.65
Number of chordaes of the anterior papillary muscle (mm)	11.7 ± 2.18
Distance anterior pillar annulus (mm)	21.9 ± 7.25
Distance anterior papillary muscle-apex (mm)	18.7 ± 5.29
Posterior papillary muscle length (mm)	30.0 ± 8.75
Number of heads of the posterior papillary muscle	1.5 ± 0.68
Number of chordaes of the posterior papillary muscle	9.6 ± 1.99
Distance posterior papillary muscle-annulus (mm)	21.0 ± 5.40
Distance posterior papillary muscle-apex (mm)	18.3 ± 4.58

Comparison of our different measured values between women and men showed no statistically significant difference (*p* > 0.05). We did not find any correlation between these different measured values (*p* > 0.05) or between age and these measured values (*p* > 0.05).

## Discussion

Dissection of these hearts and measurement was uneventful for all specimens. All dissected subjects had a mitral leaflet (anterior mitral leaflet and posterior mitral leaflet), mitral annulus, tendon chordaes, and the two papillary or pillar muscles (posteromedial pillar and anterolateral pillar). Each papillary muscle provides chordaes to each of the corresponding halves of the two valves. Comparison of our different measured values between females and males showed no statistically significant difference (*p* > 0.05). We did not find any correlation between these different measured values (*p* > 0.05) or between age and these measured values (*p* > 0.05).

For the measurement of chordaes length, we were interested in the marginal chordaes, which are also called primary chordaes. These chordaes are inserted on the free edge of the valves and have the function of opposing the prolapse of the valve leaflets. It is these chordaes that are elongated or broken when there is a prolapse of one of the valve leaflets.

This present study is interesting not only because it deals with the biometry of the annulus, the anterior and posterior valves, the chordaes and the papillary muscles of the mitral apparatus, but also because it is directly applicable to mitral valve repair surgery.

The results of this study are consistent with those found in the literature. In a series of 50 dissected hearts, Nranganathan et al. [12], found 17.5 ± 0.25 mm as the length of chordaes at the level of the anterior valve roughened area versus 17.5 ± 1.12 at the level of A1 in our series and 14.0 ± 0.08 for the length of chordaes at the level of the posterior valve roughened area versus 13.3 ± 0.83 in our series.

In a series of 20 human cadavers, Ozbag et al. [13], found an average length of the anterior papillary muscle of 33.6 ± 0.65 mm versus 30.9 ± 7.20 mm in our series and for the number of heads, he found an average of 19.6 ± 0.87 versus 1.7 ± 0.65 in our series. For the posterior papillary muscle, the average length in D. OZBAG [13], is 3.3 ± 0.64 mm, compared to 30.0 ± 8.75 mm in our series, while the number of heads is 1.9 ± 0.87 on average in OZBAG [13], compared to 1.5 ± 0.68 in our series.

In one of our recent works [14], we determined the difference between a manual, open-heart measurement and a transesophageal ultrasound (TEE) measurement of mitral valve marginal chordaes of patients who underwent valve repair surgery.

This chordae measurement was performed on 292 patients with either posterior or anterior valve prolapse or both. The sex ratio was 102 women and 190 men. The mean age was 63 years (18–81). These chordaes were first measured by TEE, immediately preoperatively, and then by they were measured by manual measurement intraoperatively. The measured distance is the distance from the tip of the papillary muscle to the free edge of the nonprolapsed valve. As a result, it was found that the two types of measurements differed by 1 mm on average in favor of the ultrasound measurement. The average of the manual measurement is 23 mm. That of the ultrasound measurement is 24 mm.

These results of manual measurement of mitral valve chordae length intraoperatively, with a stopped, hypothermic and bloodless heart are superposable to our results of mitral valve chordae length measurements in the human cadaver.

This manual intraoperative measurement, contrary to the transesophageal echographic measurement, should be reserved for patients with a contraindication to transesophageal echography: thoracic radiotherapy, esophageal pathology (stenosis, diverticulum, tumor, varices), unstable lesions of the cervical spine, massive hematemesis, evolving upper GI hemorrhagic lesion, gastric or esophageal perforation, recent esophageal surgery, situation leading to a benefit-risk assessment (severe shock, hypoxia) [15].

When replacing native chordaes with artificial chordaes during mitral plasty procedures, the surgeon is always confronted with the determination of the ideal length of the artificial chordaes. If the replacement chordaes are short, excessive tension can cause tissue rupture at the sutures. If they are very long, they cannot transmit enough tension during a cardiac cycle. Therefore, it is preferable and useful to have accurate values of the length of the native healthy marginal chordaes in order to adapt this length to the artificial neochordaes to be implanted.

The principle is to implant the neochordaes with the right length, between the tip of the head of the papillary muscle and the free edge of the valve. It is not necessary to resect the broken or elongated chorae. There is therefore no risk for secondary or tertiary chordaes if these chordaes have the same implantation on the papillary muscle.

In all cases, a homothetic reduction in a flexible ring must be systematically performed as in other plastic surgery techniques.

For this homothetic reduction, there is a relationship between our measurements and the choice of annuloplasty rings. Indeed, for each ring model, the manufacturers provide a specific sizer, which is used as a tool to determine the size of the annuloplasty ring. These sizers most often have a handle holding a D-shaped platform that includes two notches. The dimensions of the platform and the distance between the notches are used to determine the ring size. Annuloplasty rings are labeled with even (e.g., all Edwards Life sciences rings) or odd sizes (e.g., Medtronic Duran). In even rings, the distance between the sizer notches typically indicates the distance between the commissures, whereas in odd rings, the distance between the sizer notches indicates the intertrigonal distance. The platform surface is used to measure either the bare surface or the entire surface of the mitral leaflets. The septolateral diameter of the platform is used to measure the height of the anterior mitral leaflet. The quality of the annuloplasty is assessed by the coaptation height of the valve leaflets and the morphology of the closure line of these leaflets.

Nowadays, there is also the catheter-based mitral prolapse repair [16], which is very different from the method we perform in open heart. This technique consists of placing neochordaes between the free edge of the prolapsed valve and the apex of the left ventricle. This technique is performed under trans-esophageal echocardiography guidance. The equipment used is the NeoChord DS1000. During this procedure, there is no prior measurement of native chordaes. The technique requires a Trans apical approach that allows the NeoChord DS1000 to implant artificial chordaes that are made of polytetrafluoroethylene on the free edge of the prolapsed valve after grasping the valve leaflet tissue with 2 forceps. The piercing and fixation of the neochordaes is done with a special needle. After tying the two wires on the free edge of the prolapsed valve, they are directed through the interior of the ventricle to be externalized through the apex of the left ventricle, where they are tied (fixed) to a defined length for correction of the leaflet prolapse. This procedure is performed in elderly patients with a very high risk of mortality after surgery and who are most often recused from surgery.

Several authors [6, 17], have published clinical results that demonstrate that while the clinical results of triangular resection or quadrangular resection are comparable with the neo-chording technique, the latter better preserves posterior valve mobility.

Abitch et al. [18], demonstrated in a series of 171 patients who underwent mitral valve repair between January 2010 and July 2012 that the preoperative A2/P2 ratio was 1.5 ± 0.5. After repair, this A2/P2 ratio was 1.9 ± 0.3 and 2.0 ± 0.3 in the groups without plication or with partial plication of the posterior valve.

Carvalho et al. [19], studied mitral valve leaflet elongation as a phenotypic feature of cardiomyopathy. These authors report that patients with hypertrophic cardiomyopathy have a mild 5-mm elongation of the mitral valve leaflets.

The limitation of this method of chordae substitution is that it is not sufficient when it comes to Barlow's disease. In this case, the diamond resection of the excess of tissue localized in relation to the ring and the free edge of the valve, associated with the closure of slits and indentations are necessary.

We expect to find in the future, possibilities to make this technique of chordae substitution sufficient and efficient to all complex mitral prolapses including Barlow's disease. It is in this context that we should consider continuing this biometric work in the laboratory and in the operating room.

## Conclusion

The technique of replacing the native chordaes by artificial chordaes allows to reproduce the real anatomy and physiology with conservation of the movement of the two valves, thanks to a complete reconstruction of the mitral valve apparatus. A perfect mastery of anatomy and biometry is therefore essential.

## Data Availability

The datasets used and analysed during the current study are available from the corresponding author on reasonable request.
